# Anomalous intraosseous venous drainage of pretibial varices: case report

**DOI:** 10.1590/1677-5449.202400572

**Published:** 2025-03-31

**Authors:** Joana Storino, Ana Julia Resende Rocha, Ana Laura Decat Gonçalves, Luisa Reis Braga, Luma Pereira Brandão

**Affiliations:** 1 Faculdade de Ciências Médicas de Minas Gerais – FCMMG, Belo Horizonte, MG, Brasil.

**Keywords:** case report, varices, drainage, veins, tibia

## Abstract

Anomalous intraosseous venous drainage causing pretibial varices is a rare and little-known cause of varicose veins in the lower limbs (LL). We report the case of a 49-year-old male patient with chronic pain associated with large pretibial varicose veins and mild edema of the left lower limb, with no skin changes or history of previous treatment. Vascular ultrasound identified a dilatated intraosseous vein of the tibia with reflux draining to an incompetent bone perforating vein and subcutaneous varicose veins. Bone perforating veins must be carefully investigated in the presence of atypical non-saphenous and recurrent varicose veins using vascular ultrasound, which demonstrates the periosteal defect in the tibia and presence of reflux that feeds the pretibial subcutaneous varicosities. Recognizing this rare entity avoids diagnostic errors and is essential for the most appropriate treatment.

## INTRODUCTION

Varicose veins are dilated and tortuous anomalous drainage veins. They typically affect the superficial vein system of the lower limbs (LL) and have various etiologies. Lower limb varicose veins are present in 10 to 40% of people aged 30 to 70 years. Advanced age, Caucasian race and multiple gestations are important risk factors.^1^ Anomalous intraosseous venous drainage of pretibial varicose veins is a rare and little-known cause of LL varicose veins, with just 14 cases reported in the literature and greatest prevalence in male adult patients.^2^ The origin of this condition has not been fully explained, but there is evidence that it is related to prior venous insufficiency, which may be secondary (congenital anomalies) or acquired. The hypothesis has been ventured that auxiliary drainage via the intraosseous route may occur in the context of venous insufficiency.^2^

Affected individuals have unilateral chronic pain and edema of the lower limb and diagnosis is generally made late because of the complexity of the condition. An incompetent bone perforator vein and a defect of the anterior aspect of the tibia can be detected with vascular echography and recognition of the condition avoids diagnostic errors such as arteriovenous malformations and hemangiomas.^1^ Intraosseous venous drainage causes increased pressure in the peripheral venous system, culminating in venous valve incompetence. As a consequence, patients develop large varicose veins and are at increased risk of thromboembolic phenomena.^2^ The objective of this study is to report a rare case of anomalous intraosseous venous drainage with pretibial varicose veins causing chronic pain and edema in the lower limb. The medical community’s lack of recognition of this condition impairs early diagnosis and appropriate management.

The study protocol was approved by the Ethics Committee at the Faculdade de Ciências Médicas de Minas Gerais (consolidated opinion number: 6.739.905).

## CASE DESCRIPTION

**Figure 1:**
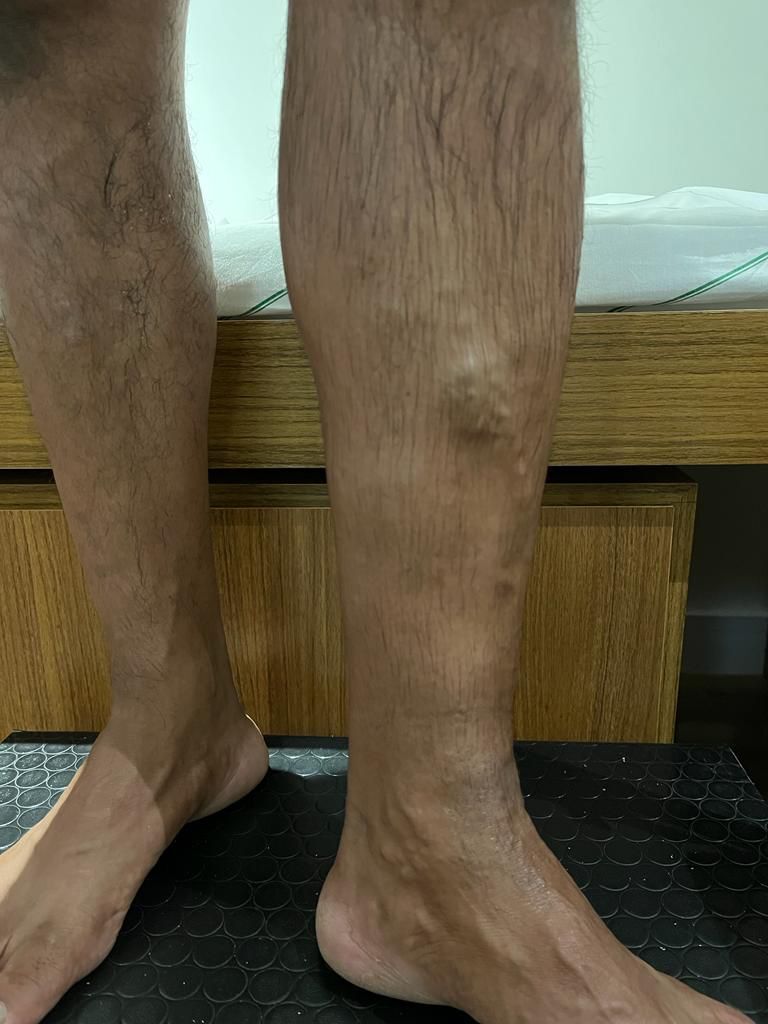
Pretibial varicose veins seen on physical examination.

The patient was a 49-year-old male postman with hypertension and chronic pain in the left pretibial area and sporadic localized swelling and edema. On physical examination, large caliber varicose veins were observed standing proud under the skin in the pretibial region, with discrete edema of the limb (Figure 1). No varicose veins were observed in other areas and there were no cutaneous changes related to chronic venous disease.

Since this is a diagnostic report, the case does not involve interventions or follow-up.

## DISCUSSION

**Figure 2:**
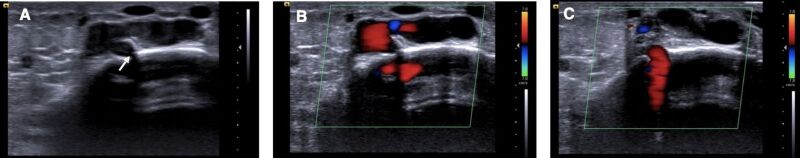
Vascular echography; (A) B-mode showing the defect in the periosteum of the tibia (white arrow); (B) presence of large caliber subcutaneous varicose veins and dilatated intraosseous veins; (C) incompetent bone perforator vein during distal compression of the leg.

**Figure 3:**
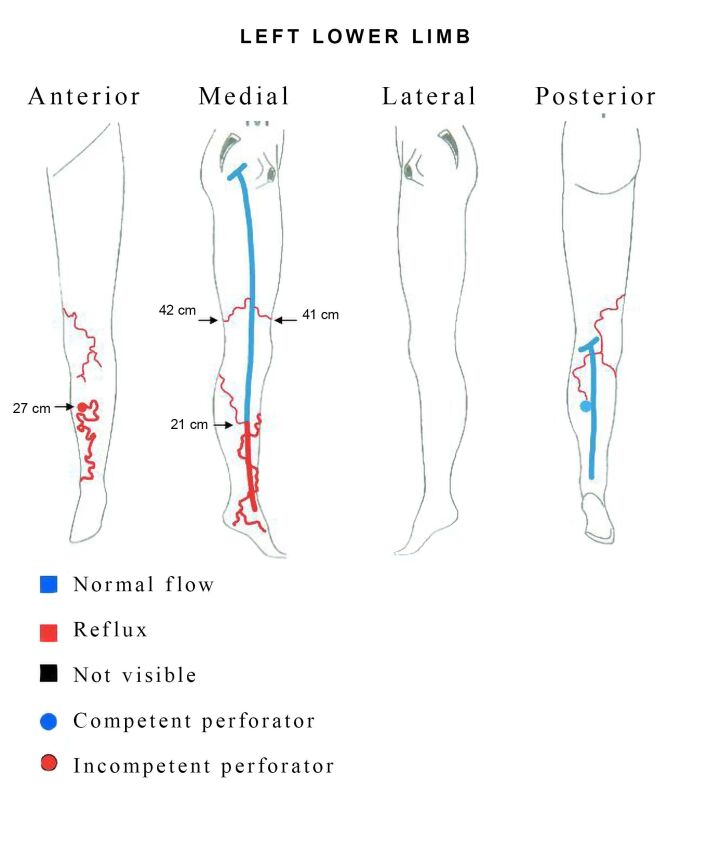
Vascular cartography of the left lower limb illustrating reflux in the distal great saphenous vein and intraosseous perforating vein with reflux related to large caliber pretibial varicose veins.

Venous duplex scan of the lower limbs showed presence of large caliber subcutaneous varicose veins in the pretibial area related to a defect in the periosteum with reflux lasting > 1 second during the distal compression maneuver (Figure 2). Reflux was observed in the distal great saphenous vein, originating in a posterior tributary, unconnected to the pretibial varicose veins. There was no reflux in the deep vein system, signs of arteriovenous malformations, or communication with the tibial veins (Figure 3). Ultrasound investigation can be enough to make a diagnosis in the majority of cases. The examination may reveal: a widened nutrient canal in the tibial diaphysis, a lytic defect observed in the anterior cortical bone subjacent to the pretibial varicose veins, a dilatated intraosseous vein, and a longitudinal sulcus in the tibial diaphysis.^1^ Recognition of these imaging findings is essential for correct diagnosis and therapeutic planning. It is extremely important that vascular surgeons are aware of this condition as an unusual cause of varicose veins in the LL.^1^

The first case reported in the literature was published in 1997 by Boutin et al.^3^ Another 13 cases have been described more recently. These patients were aged from 23 to 75 years and had unilateral changes with signs of venous insufficiency, as in the case described above.^4^

The etiology of varicose veins with anomalous intraosseous venous drainage is not yet fully understood. One hypothesis raised is that auxiliary drainage via the intraosseous route may occur in the presence of venous insufficiency.^4^

Vascularization of the tibia is primarily supplied by an arterial and venous vascular pedicle that enters a foramen via the posterior cortex of the diaphysis and meets the vessels of the epiphysis.^3^ Externally, the periosteum is richly vascularized, and blood communicates physiologically between the endosteal and periosteal networks via miniscule transosteal “perforator vessels”. We have no evidence that there are valves in these communicating veins, which are not normally involved in control of venous pressure differences. Anomalous venous drainage of the tibia may constitute a dilatation of one of these foramina, as is suggested by echography and radiography. The elevated intraosseous venous pressure may be induced by reflux in the tibial venous drainage, indicative of deep venous insufficiency.^5^

Color Doppler ultrasonography is generally the first imaging method used to investigate these lesions. This method will demonstrate a defect in the anterior diaphysis of the tibia, with typical venous reflux via the osteolytic defect in the anterior tibial cortex, feeding subcutaneous varicose veins. Doppler ultrasound may also show reflux in the posterior tibial veins and their tributaries, extending to the tibia bone.^5^ However, one disadvantage of ultrasound is that the dilatated intraosseous vein itself cannot be completely visualized, because the ultrasound waves are incapable of penetrating the bone cortex.^2^

However, magnetic resonance (MRI) may be the preferred method for confirming this anomaly of intraosseous venous drainage, because it offers superior contrast for soft tissues. Additionally, MRI can also be used to rule out other vascular anomalies that could be differential diagnoses, such as arteriovenous malformations, venous malformations, and hemangiomas.^6^

Simple radiography of the middle of the tibia is a cheap examination that is an option specifically to show a round osteolytic defect perforating the anterior cortex of the bone.

The pretibial varicose veins should be treated, especially if there are cutaneous symptoms or changes.^5^

According to the scant current literature, compression stockings are not recommended as treatment. Compression therapy works by reducing transmural pressure, by adding a source of extravascular pressure. As a result, the symptoms caused by superficial varicose veins are relieved. However, this treatment has not proven effective in cases of pretibial varicose veins with anomalous venous drainage as it is in other cases.^6^

Surgery appears to be effective in the cases that have been described. However, there is a risk of relapse years after the procedure. Sclerotherapy, with injections of ethamolin® or hypertonic glucose and ultrasound guided sclerotherapy using polidocanol foam have been employed in some of the reported cases. Depending on the case, surgical intervention procedures in combination with sclerotherapy may be recommended for treatment of varices in perforating veins. Results are varied. Several different sclerotherapy sessions^7^ or even surgical interventions may be needed to achieve satisfactory results.^2^

## CONCLUSIONS

In conclusion, we present the case of a patient with varicose veins related to an anomaly of intraosseous venous drainage, which is a rarely diagnosed disease, meaning there are few studies of the subject. Ultrasonography is generally the first imaging modality used to assess these lesions and may suffice for planning treatment. MRI is the imaging method of choice to confirm the diagnosis and rule out other vascular anomalies.
