# Supplementary material for: Anomalous intraosseous venous drainage of pretibial varices: case report
Source: J Vasc Bras. 2025 Mar 31;24:e20240057. doi: 10.1590/1677-5449.202400572 (PMC11961127; doi:10.1590/1677-5449.202400572)
Supplement: Translation

# Translation: Drenagem venosa anômala intraóssea de varizes pré-tibiais: relato de caso

**DOI:** 10.1590/1677-5449.202400571

**Authors:** Joana Storino, Ana Julia Resende Rocha, Ana Laura Decat Gonçalves, Luisa Reis Braga, Luma Pereira Brandão

**Affiliations:** 1 Faculdade de Ciências Médicas de Minas Gerais – FCMMG, Belo Horizonte, MG, Brasil.

**Keywords:** relato de caso, varizes, drenagem, veias, tíbia

## Abstract

A drenagem venosa anômala intraóssea provocando varizes pré-tibiais é uma causa rara e pouco conhecida de varizes dos membros inferiores (MMII). Relatamos o caso de um paciente do sexo masculino de 49 anos de idade com quadro de dor crônica associada a veias varicosas pré-tibiais de grosso calibre e edema discreto do membro inferior esquerdo, sem alterações cutâneas associadas ou história de tratamento prévio. À ecografia vascular, foi identificada veia intraóssea da tíbia dilatada com drenagem de refluxo para veia perfurante óssea insuficiente e varizes subcutâneas. A veia perfurante óssea deve ser cuidadosamente investigada na presença de varizes atípicas de origem não safênica e recorrentes, por meio da ecografia vascular que demonstra o defeito periósteo na tíbia associada à presença de refluxo que alimenta varicosidades subcutâneas pré-tibiais. O reconhecimento dessa rara entidade evita erros de diagnóstico e é essencial para o tratamento mais adequado.

## INTRODUÇÃO

As varizes são veias dilatadas e tortuosas de drenagem sanguínea anômala. Tipicamente, acometem mais o sistema venoso superficial dos membros inferiores (MMII) e possuem etiologias diversas. Varicosidades nos MMII estão presentes em 10 a 40% das pessoas com idade entre 30 e 70 anos. Idade avançada, raça caucasiana e múltiplas gestações são importantes fatores de risco^1^ . A drenagem venosa anômala intraóssea de varizes pré-tibiais é uma causa rara e pouco conhecida de varizes nos MMII, havendo apenas 14 casos reportados na literatura, com maior prevalência em pacientes adultos do sexo masculino^2^ . A origem dessa condição ainda não foi completamente elucidada, mas há evidências de que se relaciona a insuficiência venosa prévia, secundária (anomalias congênitas) ou adquirida. Foi levantada a hipótese de que uma drenagem auxiliar, pela via intraóssea, pode ocorrer no cenário da insuficiência venosa^2^ .

Os indivíduos acometidos cursam com dor crônica unilateral do membro inferior e edema, e o diagnóstico geralmente é tardio devido a sua complexidade. A presença de veia perfurante óssea incompetente e defeito na face anterior da tíbia pode ser detectada por ecografia vascular, e seu reconhecimento evita erros de diagnóstico, como malformações arteriovenosas e hemangiomas^1^ . A drenagem venosa intraóssea leva a um aumento da pressão no sistema venoso periférico que culmina com insuficiência das válvulas venosas. Como consequência, os pacientes apresentam varizes volumosas e um risco aumentado para fenômenos tromboembólicos^2^ . O objetivo deste trabalho é relatar um caso raro de drenagem venosa anômala intraóssea com varizes pré-tibiais que cursam com dor crônica e edema de membro inferior. A falta de reconhecimento dessa condição pela comunidade médica prejudica o diagnóstico precoce e o manejo adequado do caso.

O protocolo foi aprovado pelo Comitê de Ética da Faculdade de Ciências Médicas de Minas Gerais (número do parecer consubstanciado: 6.739.905).

## DESCRIÇÃO DO CASO

Paciente do sexo masculino, 49 anos, carteiro, hipertenso, com quadro de dor crônica em região pré-tibial esquerda e abaulamento no local com edema esporádico. Ao exame físico, observaram-se veias varicosas de grosso calibre saltadas na pele em região pré-tibial e edema discreto do membro (Figura 1). Não foram observadas veias varicosas em outras regiões e alterações cutâneas relacionadas à doença venosa crônica.

**Figura 1:** Varizes pré-tibiais ao exame físico.

Por se tratar de um relato referente ao diagnóstico, não foram realizadas intervenções ou acompanhamento neste caso.

## DISCUSSÃO

Ao *duplex scan* venoso dos membros inferiores, evidenciou-se a presença de varizes calibrosas subcutâneas em região pré-tibial associadas a falha em periósteo com refluxo com duração > 1 segundo durante a manobra de compressão distal (Figura 2). Foi observado refluxo distal de veia safena magna a partir de tributária posterior, sem conexão com as varizes pré-tibiais. Não houve refluxo no sistema venoso profundo, sinais de malformação arteriovenosa ou comunicação com as veias tibiais (Figura 3). A investigação ecográfica pode ser suficiente para o diagnóstico na maioria dos casos. Durante o exame, podem ser identificados: canal nutrício alargado na diáfise tibial, defeito lítico observado na cortical anterior subjacente às varizes pré-tibiais, veia intraóssea dilatada e sulco longitudinal na diáfise tibial^1^ . O reconhecimento desses achados de imagem é fundamental para o correto diagnóstico e planejamento terapêutico. É de extrema importância que os cirurgiões vasculares tenham conhecimento dessa condição como uma causa não usual de varizes nos MMII^1^ .

**Figura 2:** Ecografia vascular. (A) modo b evidenciando a falha no periósteo da tíbia (seta branca); (B) presença de varizes subcutâneas calibrosas e veias intraósseas dilatadas; (C) veia perfurante óssea insuficiente durante compressão distal da perna.

**Figura 3:** Cartografia vascular do membro inferior esquerdo demonstrando refluxo distal de veia safena magna e perfurante intraóssea com refluxo associando-se a varizes calibrosas pré-tibiais.

O primeiro caso relatado na literatura foi publicado em 1997 por Boutin et al.^3^ . Recentemente, foram descritos mais 13 casos. Os pacientes possuíam entre 23 e 75 anos e alterações unilaterais com sinais de insuficiência venosa, assim como o paciente relatado^4^ .

A etiologia das varizes com drenagem venosa anômala intraóssea ainda não é plenamente compreendida. Foi levantada a hipótese de que uma drenagem auxiliar, pela via intraóssea, pode ocorrer na insuficiência venosa^4^ .

A vascularização da tíbia é suprida principalmente por um pedículo vascular arterial e venoso, que penetra em um forame através do córtex posterior da diáfise e se une aos vasos da epífise^3^ . Externamente, o periósteo é ricamente vascularizado, sendo que o sangue se comunica fisiologicamente entre as redes endosteal e periosteal através de minúsculos “vasos perfurantes” transosteais. Não temos evidências da presença de válvulas nessas veias comunicantes, que normalmente não estão envolvidas no controle da diferença de pressão venosa. A drenagem venosa anômala da tíbia pode corresponder a uma dilatação de um desses forames, como sugerido por ecografia e radiografia. A hiperpressão venosa intraóssea pode ser induzida por um refluxo na drenagem venosa da tíbia, indicativo de insuficiência venosa profunda^5^ .

A ultrassonografia com Doppler colorido geralmente é o primeiro método de imagem a ser usado para avaliar a lesão. Esse método demonstra o defeito da diáfise anterior da tíbia, com refluxo venoso típico através do defeito osteolítico do córtex tibial anterior, alimentando varizes subcutâneas. O Doppler também pode demonstrar refluxo nas veias tibiais posteriores e suas tributárias estendendo-se para o osso da tíbia^5^ . Uma desvantagem do ultrassom, no entanto, é que a própria veia intraóssea dilatada não pode ser completamente visibilizada, devido à incapacidade das ondas de ultrassom em penetrar no córtex ósseo^2^ .

No entanto, a ressonância magnética (RM) pode ser o método preferido para confirmar essa anomalia da drenagem venosa intraóssea, por oferecer contraste superior de tecidos moles. Além disso, a RM também pode ser usada para descartar outras anomalias vasculares que poderiam ser diagnósticos diferenciais, como malformações arteriovenosas, malformações venosas e hemangiomas^6^ .

A radiografia simples do meio da tíbia é um exame econômico e com abordagem específica para demonstrar um defeito osteolítico redondo perfurando o córtex anterior do osso.

As varizes pré-tibiais devem ser tratadas especialmente se apresentarem sintomas ou alterações cutâneas^5^ .

De acordo com a escassa literatura atual, meias de compressão não são recomendadas como tratamento. A terapia de compressão atua através da diminuição da pressão transmural, por meio de adição de uma pressão extravascular. Dessa forma, os sintomas causados pelas varizes superficiais são aliviados. Entretanto, em casos de varizes pré-tibiais com drenagem venosa anômala, o tratamento não se demonstra efetivo como nos demais casos^6^ .

A cirurgia aparenta ser eficaz nos casos descritos. Contudo, existe o risco de recidiva anos após o procedimento. A escleroterapia, por meio da injeção de ethamolin® ou glicose hipertônica, e a escleroterapia guiada por ultrassom, através de espuma de polidocanol, foram utilizadas em alguns casos relatados. A depender do caso, os procedimentos de intervenção cirúrgica em combinação com a escleroterapia são recomendados no tratamento de varicosidades em veias perfurantes. Os resultados são variados. Podem ser necessárias diversas sessões de diferentes escleroterapias^7^ ou até mesmo de intervenções cirúrgicas para obter resultados satisfatórios^2^ .

## CONCLUSÃO

Em conclusão, apresentamos o caso de um paciente com varizes relacionadas a anomalia de drenagem venosa intraóssea, que se caracteriza como uma doença de diagnóstico raro, havendo, portanto, poucos estudos sobre o tema. A ultrassonografia é geralmente a primeira modalidade de imagem para avaliar a lesão e pode ser suficiente para o planejamento terapêutico. A RM é a modalidade de imagem de escolha para confirmar o diagnóstico e descartar outras anomalias vasculares.
